# Overcoming Chemotherapy Resistance in Triple-Negative Breast Cancer with Nanocarrier-Delivered siRNA Therapeutics

**DOI:** 10.3390/jcm15062311

**Published:** 2026-03-18

**Authors:** Andreea Crintea, Corina I. Bocșan, Elena M. Jianu, Alina S. Șovrea, Camelia Munteanu, Milan P. Kubelac, Alexandra M. Crăciun, Ciprian N. Silaghi

**Affiliations:** 1 Medical Biochemistry, Department of Molecular Sciences, “Iuliu Haţieganu” University of Medicine and Pharmacy, 400349 Cluj-Napoca, Romania; crintea.andreea@umfcluj.ro (A.C.); silaghi.ciprian@umfcluj.ro (C.N.S.); 2 Pharmacology, Toxicology and Clinical Pharmacology, Department of Morpho-Functional Sciences, “Iuliu Haţieganu” University of Medicine and Pharmacy, 400012 Cluj-Napoca, Romania; bocsan.corina@umfcluj.ro; 3 Histology Section, Department of Morpho-Functional Sciences, “Iuliu Haţieganu” University of Medicine and Pharmacy, 400012 Cluj-Napoca, Romania; marina.elena@umfcluj.ro (E.M.J.); sovrea@umfcluj.ro (A.S.Ș.); 4 Biology Section, University of Agricultural Sciences and Veterinary Medicine Cluj-Napoca, 400372 Cluj-Napoca, Romania; camelia.munteanu@usamvcluj.ro; 5 Faculty of Medical and Health Sciences, Babeș-Bolyai University, 400349 Cluj-Napoca, Romania; milan.kubelac@ubbcluj.ro; 6 Institute of Oncology “Prof. Dr. Ion Chiricuță”, 400015 Cluj-Napoca, Romania

**Keywords:** triple-negative breast cancer, siRNA, nanocarriers, nanoparticles

## Abstract

Triple-negative breast cancer (TNBC) represents 10–20% of breast cancers and is characterized by the absence of estrogen receptor, progesterone receptor, and human epidermal growth factor receptor 2 expression, leaving cytotoxic chemotherapy as the main systemic treatment. However, rapid development of resistance, via drug efflux, enhanced DNA repair, apoptosis evasion, epithelial-to-mesenchymal transition, and tumor microenvironment protection, limit long-term efficacy. Small interfering RNA (siRNA) therapeutics can silence key resistance drivers, but their clinical potential is hindered by instability, poor biodistribution, and off-target effects. Nanocarrier-based delivery systems offer solutions by protecting siRNA, enhancing tumor accumulation, enabling targeted intracellular release, and permitting co-delivery with chemotherapeutics for synergistic effects. We conducted a narrative review in PubMed from database inception to August 2025. The included studies demonstrated that lipid, polymeric, inorganic, and hybrid nanocarriers can achieve efficient target knockdown, reverse drug resistance mechanisms, and significantly enhance antitumor responses in resistant TNBC models. Several platforms also reduced metastatic spread and improved survival in vivo. While preclinical results are compelling, clinical translation remains limited by incomplete safety profiling and heterogeneity in delivery efficiency. This review synthesizes mechanistic insights and delivery innovations, outlining a roadmap for translating siRNA-loaded nanocarriers into effective therapies for chemoresistant TNBC.

## 1. Introduction

Triple-negative breast cancer (TNBC) accounts for approximately 10–20% of all breast cancer cases. This subtype is defined by the absence of estrogen receptor (ER), progesterone receptor (PR), and human epidermal growth factor receptor 2 (HER2) expression, factors that render both endocrine and HER2-targeted therapies ineffective [1,2,3,4]. Clinically, TNBC is aggressive, with early onset, rapid metastatic spread, and a propensity for early relapse, leading to poorer long-term survival compared to other subtypes [1,5]. The precise definition of TNBC, ER-negative, PR-negative, HER2-negative, aligns with standard molecular classification guidelines and is consistently used across epidemiologic and clinical studies [6,7,8].

This receptor-negative phenotype eliminates the use of endocrine and HER2-targeted therapies, leaving cytotoxic chemotherapy, most commonly taxanes, anthracyclines, and platinum-based agents, as the current mainstay of systemic treatment [9].

Although initial responses to chemotherapy can be favorable, they are frequently short-lived, with relapse rates remaining high and overall survival limited [10]. This chemoresistance is mediated by multiple, often concurrent mechanisms. Overexpression of ATP-binding cassette (ABC) transporters, such as multidrug resistance-associated protein 1 (MRP1, or ABCC1), reduces intracellular drug concentrations [11]. Enhanced DNA repair capacity, exemplified by radiation-sensitive protein 51 (RAD51) overexpression, facilitates tolerance to DNA-damaging agents [12,13]. Anti-apoptotic signaling through molecules such as B-cell lymphoma-2 (BCL-2) [14] and the inhibitor of nuclear factor kappa-B kinase epsilon (IKBKE) [15] promotes tumor cell survival under chemotherapeutic stress. In parallel, epithelial-to-mesenchymal transition (EMT) programs enhance invasiveness and contribute to drug resistance [16].

The tumor microenvironment (TME) adds further complexity, with cancer-associated fibroblasts, extracellular matrix remodeling, and hypoxic niches providing physical and biochemical protection, while immune-suppressive signaling dampens anti-tumor immunity [17,18]. These multifactorial barriers underscore the urgent need for targeted strategies that can modulate specific resistance pathways without harming normal tissues.

Small interfering RNA (siRNA) therapeutics offer a compelling approach by enabling sequence-specific silencing of genes driving chemoresistance [19,20,21]. In TNBC, siRNAs can be designed to inhibit targets such as drug efflux pumps [22], DNA repair enzymes [13], anti-apoptotic regulators [23,24], and EMT transcription factors [25,26], directly resensitizing tumor cells to conventional chemotherapy [27]. However, naked siRNA is inherently unstable in circulation, prone to enzymatic degradation, immune activation, and rapid renal clearance, with inefficient tumor uptake [19,28].

A variety of nanocarrier platforms have been developed to facilitate siRNA delivery, each offering distinct structural and functional advantages. Lipid nanoparticles (LNPs) remain the most clinically advanced approach, enabling efficient encapsulation and endosomal escape through ionizable lipid components [21,29]. Polymeric carriers, including cationic polymers, dendrimers, and block copolymers, offer customizable structures and stimuli-responsive designs but may require careful optimization to limit cytotoxicity [30]. Inorganic platforms such as mesoporous silica, gold nanoparticles, or magnetic nanomaterials provide high loading capacity and opportunities for imaging or theranostic applications [31]. Hybrid systems combine organic and inorganic components to balance stability, targeting specificity, and controlled release [32].

Within TNBC research, these nanocarrier systems have been adapted to protect siRNA, enhance tumor accumulation, facilitate cellular uptake, and enable controlled intracellular release [19,28]. Platforms explored in TNBC include LNPs [33], polymer-based carriers [34], inorganic nanoparticles [24,35], and hybrid [23] or stimuli-responsive systems [21]. Beyond improving pharmacokinetics, nanocarriers can be functionalized with targeting ligands [24,36,37], exploit tumor-specific triggers (e.g., pH, enzymes, ultrasound) [36,38,39], and co-deliver chemotherapeutics and siRNAs in a single construct to achieve synergistic reversal of drug resistance [22,33]. In this context, an evaluation of nanocarrier platforms for siRNA delivery in TNBC is warranted to identify the most promising strategies, delineate their mechanistic advantages, and guide the rational design of future therapeutic systems capable of overcoming chemotherapy resistance.

In recent years, several siRNA-based therapeutics have reached clinical approval, offering useful context for the delivery strategies discussed in this review. Patisiran, formulated in lipid nanoparticles, was the first RNAi drug to demonstrate that systemic siRNA delivery can be both effective and safe in humans [40], while the GalNAc-conjugated agents inclisiran, givosiran and lumasiran have further shown that reproducible gene silencing with manageable safety profiles is feasible in routine clinical care [41,42,43]. Early clinical studies of nanoparticle-based siRNA formulations in solid tumors, such as the cyclodextrin polymer system CALAA-01 and the liposomal therapeutic Atu027, have reported variable tolerability and modest tumor uptake, highlighting the ongoing challenges of translating these technologies to oncology [44,45]. Together, these clinical precedents help frame the preclinical TNBC nanocarrier data in a broader therapeutic landscape and illustrate both the promise and the remaining challenges for bringing siRNA delivery systems closer to clinical use.

## 2. Molecular Basis of Cancer for siRNA Gene Therapy

### 2.1. Oncogene Activation, Tumor Suppressor Inactivation, and Epigenetic Shifts

The initiation and progression of cancer are fundamentally driven by alterations in genes that regulate cell growth, division, and death. These alterations primarily affect two broad classes: oncogenes and tumor suppressor genes (TSGs) [46]. Oncogenes arise from proto-oncogenes through activating point mutations, gene amplification, or chromosomal rearrangements, leading to constitutive activation of signaling pathways that drive malignant proliferation. Prototypical examples include rat sarcoma (*RAS*) mutations, which sustain proliferative signaling through the mitogen-activated protein kinase (MAPK) and phosphatidylinositol 3-kinase (PI3K) cascades [46,47], and myelocytomatosis (*MYC*) amplifications, which enhance cell cycle progression and metabolic reprogramming [46,48]. Cooperative interactions between oncogenes, such as *RAS* and *MYC*, can synergistically accelerate tumorigenesis, while anti-apoptotic oncogenes such as *BCL2* confer survival advantages by suppressing programmed cell death [46,49].

In contrast, TSGs normally constrain cell proliferation and promote apoptosis. Their loss through mutation, deletion, or epigenetic silencing removes these safeguards, enabling malignant transformation. Retinoblastoma 1 (*RB1*) and tumor protein p53 (*TP53*) are among the most frequently disrupted TSGs in human cancers [46]; in particular, *TP53* dysfunction is nearly ubiquitous in triple-negative breast cancer and contributes to therapy resistance by impairing DNA damage responses [50,51,52]. Beyond direct mutations, epigenetic alterations play a pivotal role in tumorigenesis. Aberrant DNA methylation, histone modifications, and dysregulated non-coding RNAs can silence TSGs or aberrantly activate oncogenes [53,54]. Importantly, these changes are potentially reversible, making them attractive therapeutic targets. The siRNA technology is uniquely positioned in this context: by selectively silencing oncogenic drivers or regulators of epigenetic repression, siRNA can counteract oncogene overexpression and, indirectly, restore tumor suppressor function [55,56].

### 2.2. siRNA Against Drug Resistance and Undruggable Targets

Chemotherapy resistance is one of the most pressing challenges in the management of TNBC, limiting the durability of clinical responses and contributing to poor overall survival [9,57]. This resistance arises from a complex interplay of molecular mechanisms that together enable tumor cells to withstand cytotoxic stress [58].

A well-characterized mechanism is the overexpression of ABC transporters, including ATP-binding cassette sub-family B member 1 or multidrug resistance protein 1 (*ABCB1* or *MDR1*) and ATP-binding cassette subfamily G member 2 (*ABCG2*), which actively export chemotherapeutic agents and thereby reduce their intracellular concentrations [59]. In TNBC, elevated *MDR1* expression has been directly associated with resistance to paclitaxel and doxorubicin [60]. Preclinical studies demonstrate that siRNA-mediated silencing of *MDR1* restores intracellular drug retention and resensitizes resistant TNBC cells to these agents, underscoring its therapeutic promise [20,61].

DNA repair pathways also contribute substantially to resistance. TNBC frequently exhibits upregulation of *RAD51*, which facilitates homologous recombination repair and mediates resistance to DNA-damaging agents, even in tumors lacking breast cancer gene 1 and 2 (*BRCA1* and *BRCA2*) mutations [62]. Similarly, high levels of poly(ADP-ribose) polymerase 1 (*PARP1*) and excision repair cross-complementation group 1 (*ERCC1*) correlate with poor responses to platinum compounds and taxanes [63]. The siRNA knockdown of these genes has been shown to enhance cisplatin and docetaxel efficacy in preclinical cancer models [64], highlighting RNAi’s ability to undermine DNA repair–driven chemoresistance.

Evasion of apoptosis provides another layer of protection. Anti-apoptotic proteins such as *BCL2* and myeloid cell leukemia-1 (*MCL1*) are frequently overexpressed in chemoresistant TNBC, enabling tumor cells to survive despite extensive DNA damage [65,66,67]. The siRNA-mediated depletion of these proteins restores apoptotic sensitivity and amplifies the cytotoxic effects of chemotherapy [68,69,70]. In parallel, oncogenic signaling pathways, most notably the phosphatidylinositol-3-kinase/protein kinase B/mechanistic target of rapamycin (PI3K/AKT/mTOR) axis, aberrantly activated in up to 40% of TNBC cases [58], and Kirsten rat sarcoma viral oncogene homolog (KRAS)-driven cascades, promote unchecked proliferation and survival. Preclinical studies combining siRNA against PI3K/AKT/mTOR or *KRAS* with epidermal growth factor receptor (EGFR) inhibitors have demonstrated synergistic antitumor effects, suggesting translational opportunities for combinatorial therapy [25,71].

Importantly, siRNA therapeutics can also address oncogenic drivers that have historically been resistant to conventional drug development [72]. Many TNBC drivers lack suitable binding pockets for small molecules or harbor mutations that impair drug binding [58]. Unlike conventional drugs that act at the protein level, siRNAs intervene upstream at the mRNA level, preventing protein synthesis irrespective of mutation or amplification status [56]. This “supply-side” blockade directly reduces oncogenic protein load and provides a uniquely versatile strategy to counter mechanisms that remain inaccessible to antibody- or small molecule–based therapies [25].

Together, these insights highlight why siRNA represents a particularly attractive modality for TNBC: it can selectively disrupt multiple, often concurrent resistance mechanisms, resensitize tumors to chemotherapy, and expand the therapeutic arsenal to targets previously deemed intractable.

## 3. RNA Interference and the Precision of siRNA

RNA interference (RNAi) is an evolutionarily conserved mechanism of sequence-specific gene silencing mediated by small RNAs [19,27,55]. In therapeutic applications, siRNA is typically designed as a 19–21 nucleotide duplex that, once internalized by the cell, harnesses the endogenous RNAi machinery to selectively suppress gene expression [19,55].

The process begins when double-stranded RNA is recognized and cleaved by the RNase III enzyme Dicer into siRNA fragments. These are then loaded into the RNA-induced silencing complex (RISC), where the passenger strand is degraded while the guide strand is incorporated into Argonaute-2 (Ago2), the catalytic core of the complex. Guided by base-pair complementarity, Ago2 directs RISC to complementary messenger RNA (mRNA) sequences. When near-perfect alignment occurs, Ago2 cleaves the mRNA, which is subsequently degraded by exonucleases, effectively preventing translation and reducing the abundance of the encoded protein [19,21].

This endogenous pathway confers a high degree of specificity and control. Sequence complementarity functions as an intrinsic quality-control mechanism, minimizing off-target effects, while the partial knockdown of target expression, rather than complete gene ablation, preserves cellular viability [27]. These features make siRNA particularly well-suited for therapeutic applications in TNBC, where resistance arises from the simultaneous activation of multiple pathways [18,21,27,58]. By enabling precise, modular, and combinatorial suppression of distinct resistance drivers, siRNA offers a rational approach to achieving synergistic sensitization of tumors to chemotherapy.

Despite advances in molecular oncology and the emergence of RNAi as a versatile therapeutic modality, its application in TNBC remains at an early stage. The objective of this review is to compare the biological performance, safety, and translational readiness of different nanocarrier classes, including lipid, polymeric, inorganic, and hybrid systems, specifically within the TNBC setting. This review aims to provide a comprehensive assessment of their potential, highlight existing limitations, and propose a research roadmap to accelerate the development of clinically viable siRNA nanotherapeutics for drug-resistant TNBC.

## 4. Materials and Methods

A systematic search was performed in PubMed from database inception to 15 August 2025 to identify primary experimental studies investigating nanocarrier-mediated siRNA delivery for overcoming chemotherapy resistance in TNBC. The search combined terms related to TNBC, siRNA, nanocarriers, and drug resistance, using Boolean operators to capture variations in terminology. Representative queries included combinations such as (“Triple-Negative Breast Cancer” OR “TNBC”) AND (“Chemotherapy Resistance” OR “Drug Resistance”) AND (“siRNA” OR “RNA interference”) AND (“Nanocarrier*” OR “Nanoparticl*”). No language restrictions were applied at the search stage to minimize selection bias. However, because the extraction of experimental design, dosing, endpoints, and resistance validation requires full methodological detail, only records with accessible full text in English were eligible for inclusion.

Studies were included if they (i) reported primary in vitro and/or in vivo experiments using TNBC models; (ii) delivered siRNA using an engineered nanocarrier (lipid, polymeric, inorganic, hybrid, or extracellular vesicle-derived); and (iii) evaluated outcomes relevant to chemotherapy resistance (e.g., re-sensitization to a cytotoxic drug, modulation of resistance pathways, or resistance-linked phenotypes). Records without an accessible English full text were excluded. Reviews, meta-analyses, commentaries, conference abstracts, studies without TNBC models, those delivering naked siRNA without a nanocarrier, and those lacking resistance-related endpoints were also excluded.

Two reviewers independently screened titles and abstracts, with discrepancies resolved by discussion. Full texts of potentially eligible articles were assessed against the inclusion criteria, and reasons for exclusion were recorded. For each study included, data were extracted on authorship, year of publication, nanocarrier type and composition, siRNA target, experimental model, chemotherapeutic co-administration (if applicable), and main outcomes related to drug resistance. Due to heterogeneity in study designs, molecular targets, and outcome measures, a narrative synthesis was undertaken. Studies were stratified into two categories: Group 1, comprising in vivo chemotherapy re-sensitization studies in which siRNA nanocarriers were co-administered with chemotherapeutics in resistant TNBC models, and Group 2, comprising mechanistic studies that targeted resistance pathways without in vivo chemotherapy co-administration. Each in vivo study was assessed for reporting of: (1) randomization, (2) allocation concealment, (3) blinding of intervention and/or outcome assessment, (4) sample size justification or power calculation, (5) completeness of outcome reporting (*n* per group, variance measures, statistical testing), (6) reporting of exclusions or attrition, (7) validation of the chemotherapy-resistant phenotype when “resistance” or “re-sensitization” was claimed, and (8) model relevance (orthotopic implantation and immune status description).

Each domain was classified as reported, unclear, or not reported. Because of heterogeneity in design and endpoints, no numeric scoring system was applied. The appraisal was used to inform the strength of conclusions in the narrative synthesis. In addition to nanocarrier composition and siRNA target, we extracted key methodological descriptors to support interpretability, including: (i) resistance induction and validation criteria (e.g., IC_50_ shift, resistant subline, in vivo non-response to standard therapy); (ii) tumor model characteristics (cell line, orthotopic vs. ectopic implantation, syngeneic vs. xenograft); (iii) mouse strain and immune status; (iv) dosing schedules for siRNA and chemotherapeutic agents (dose, route, frequency); (v) primary endpoints and timepoints; and (vi) reported safety assessments. These variables were synthesized narratively due to heterogeneity in reporting.

## 5. Results

**Figure 1:**
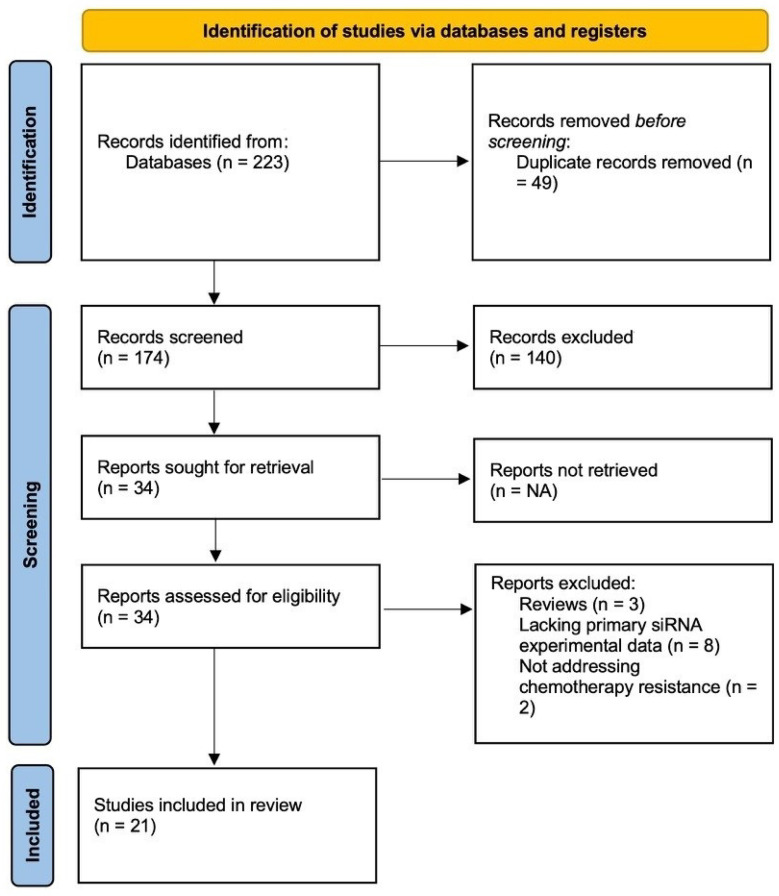
Flow diagram illustrating the study selection process for inclusion in the review.

The search yielded 174 records after removal of duplicates. Screening of titles and abstracts led to the exclusion of 32 records for irrelevance, leaving 142 articles for further evaluation. Of these, 108 were excluded for reasons including lack of focus on TNBC, absence of siRNA-based nanocarriers, or reliance on non-engineered delivery systems. Thirty-four articles underwent full-text review, with 13 excluded due to being reviews, lacking primary siRNA experimental data, or not addressing chemotherapy resistance. Ultimately, 21 studies fulfilled all inclusion criteria and were incorporated into the qualitative synthesis according to Figure 1.

To address the aim of evaluating the role of siRNA-loaded nanocarriers in overcoming TNBC chemoresistance, studies were stratified into two groups based on their translational endpoint. Group 1 (*n* = 8) comprised in vivo chemotherapy combination studies, in which siRNA nanocarriers were co-administered with standard chemotherapeutics in TNBC models. Because resistance validation was inconsistent across these reports, the extent to which individual studies represent true chemo-re-sensitization versus enhanced combination efficacy is indicated in Table 1. Group 2 (*n* = 13) comprised mechanistic and synergistic studies that did not include in vivo chemotherapy co-administration, including in vitro chemo-combination studies and pathway-targeting studies without in vivo chemotherapy. Therefore, Group 2 represents mechanistic studies rather than confirmed chemo-resistant models, and their findings should not be interpreted as evidence of in vivo chemo-re-sensitization.

Reporting of internal validity measures was heterogeneous. Among the in vivo studies (*n* = 15), explicit randomization was reported in 3 (20%), while blinding of outcome assessment was described in 1 (7%). Allocation concealment was not reported. Sample size justification or formal power calculations were not described.

Validation of chemotherapy-resistant phenotypes was clearly documented in 6 of the 21 included studies (29%), partially described in 3, and not formally demonstrated in the remaining studies that nonetheless referred to “re-sensitization”.

Orthotopic implantation models were used in 8 of 15 in vivo studies (53%), whereas the remainder relied on ectopic xenografts. The immune status of the experimental animals was reported in all in vivo experiments.

Variance measures and statistical testing were reported in most studies; however, standardized effect sizes and confidence intervals were rarely provided, limiting cross-study comparability. Overall, the evidence base demonstrates strong mechanistic exploration but variable reporting of bias-mitigating measures.

Most in vivo studies employed human TNBC cell-line xenografts, with orthotopic implantation reported in approximately half of experiments. Mouse strains were typically immunodeficient (e.g., nude or NSG), and immune status was generally specified.

Resistance induction strategies varied and included established drug-resistant sublines (e.g., 4T1/PTX), prolonged in vitro drug exposure prior to implantation, or inference based on pathway overexpression. However, formal validation through sustained IC_50_ shifts or documented in vivo non-response to standard chemotherapy was inconsistently reported.

Dosing regimens for siRNA nanocarriers and chemotherapeutics varied substantially in dose, route (intravenous or intratumoral), and frequency, limiting cross-study comparability. Primary endpoints most commonly included tumor volume inhibition, with fewer studies reporting survival or metastatic burden. Safety assessments were generally limited to body weight monitoring, with detailed hematologic or biochemical analyses rarely described.

In this review, chemoresistance refers to the intrinsic or acquired reduction in tumor sensitivity to chemotherapeutic agents, typically arising from mechanisms such as drug efflux, enhanced DNA repair, apoptotic evasion, or EMT-driven changes. Chemo-re-sensitization is defined as the restoration or enhancement of chemotherapy response through molecular, genetic, or delivery-based interventions, including siRNA-mediated targeting of resistance pathways.

### 5.1. Group 1: In Vivo Chemotherapy Re-Sensitization

Eight studies investigated the ability of siRNA-loaded nanocarriers to restore chemosensitivity in TNBC models through co-administration with standard cytotoxic agents (Table 1). Across these reports, combination strategies consistently achieved superior tumor control compared with either modality alone, highlighting diverse but complementary mechanisms of resistance reversal.

**Figure 2:**
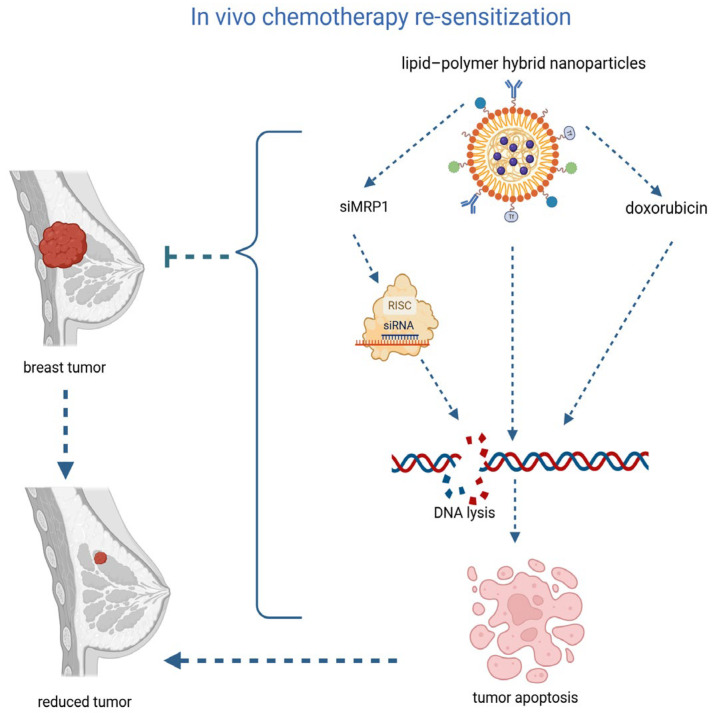
In vivo chemotherapy re-sensitization using lipid–polymer hybrid nanoparticles co-delivering doxorubicin and siMRP1. siMRP1-mediated silencing of the efflux transporter MRP1 via RISC enhances intracellular doxorubicin accumulation, leading to increased DNA damage, tumor cell apoptosis, and overall tumor regression.

Several strategies focused on efflux transporters and survival pathways, directly restoring anthracycline or taxane activity. Layer-by-layer lipid–polymer hybrid nanoparticles co-delivering doxorubicin with siMRP1 achieved approximately 80% knockdown of *MRP1* in a specific human TNBC cell line (MDA-MB-231) implanted into an immunodeficient mouse (xenografts) and produced an approximately 8-fold reduction in tumor volume relative to doxorubicin alone (Figure 2) [22]. These preclinical data indicate that suppression of efflux transporters may help restore anthracycline activity, though confirmation in standardized resistant models and in vivo systems is still needed. Redox-sensitive hyaluronic acid micelles encapsulating paclitaxel and aurora kinase A (AURKA) specific siRNA (siAURKA) displayed glutathione-triggered release, efficient tumor accumulation, and significantly greater tumor growth inhibition than either component alone. Tumor volume was suppressed by nearly 70% compared with doxorubicin alone, highlighting mitotic kinase inhibition as an effective lever for sensitization [38]. A multistage porous-silicon microvector system encapsulating siRAD51 and doxorubicin reduced primary tumor burden by approximately 65% and curtailed pulmonary metastases in orthotopic models, with marked decreases in metastatic foci compared with doxorubicin alone [13]. These data underscore the potential of targeting homologous recombination to extend benefits beyond local control.

In an orthotopic TNBC xenograft model, hyaluronic acid–modified hybrid nanocomplexes carrying siIKBKE with cabazitaxel significantly enhanced apoptosis, reduced invasiveness, and prolonged survival compared to either agent alone [36]. In a resistant setting, dendritic polylysine nanoparticles co-delivering siRNA targeting the AXL (tyrosine kinase receptor) gene (siAXL) and paclitaxel, reversed paclitaxel refractoriness in an orthotopic paclitaxel-resistant 4T1 mouse mammary carcinoma cell line treated with paclitaxel (4T1/PTX), restoring sensitivity and producing substantial tumor regression compared with paclitaxel-alone treatment, which was ineffective in the resistant mode [34].

Two studies converged on autophagy inhibition as a sensitization axis. CL4-aptamer–engineered exosomes delivering aspartyl-tRNA synthetase antisense RNA 1 siRNA (siDARS-AS1) with doxorubicin, reversed anthracycline resistance via blockade of transforming growth factor-β/Sma and Mad 3 (TGF-β/Smad3)-mediated autophagy, reducing tumor growth by approximately 60% compared with doxorubicin alone [74]. Polymeric “smart” nanoparticles carrying microtubule-associated protein 1 light chain 3 siRNA (siLC3) similarly reinstated doxorubicin efficacy, with combination treatment yielding significantly greater inhibition of proliferation, migration, and colony formation in vitro and translating into superior tumor growth suppression in vivo [75].

Cluster of differentiation 44 (CD44)-aptamer decorated extracellular vesicles co-delivering survivin siRNA with gemcitabine or paclitaxel enabled marked dose reductions (more than 50%) of chemotherapy while maintaining tumor inhibition. Systemic toxicity was lower compared with full-dose chemotherapy controls [73], offering a clinically relevant advantage.

These studies demonstrate that siRNA nanocarriers can restore chemotherapy responsiveness in TNBC through diverse, non-redundant mechanisms, including efflux blockade, checkpoint disruption, DNA repair inhibition, factor nuclear kappa B (NF-κB) and AXL suppression, and autophagy targeting. Benefits extended beyond tumor shrinkage to include survival gains, reduced metastasis, and chemotherapy dose minimization, pointing to substantial translational promise. However, systematic long-term toxicology and biodistribution analyses remain sparse, representing a key gap for clinical advancement.

### 5.2. Group 2: Mechanistic Nanocarrier–siRNA Studies Without In Vivo Chemotherapy

Thirteen studies evaluated siRNA nanocarriers that achieved robust gene silencing and antitumor effects in TNBC but did not combine siRNA delivery with standard chemotherapeutics in vivo (Table 2). Antibody-decorated nanoshells that deliver catenin beta 1 (CTNNB1) siRNA reduced primary growth, lung metastasis, and recurrence—an in vivo validation of Wingless and int-1/β-catenin (Wnt/β-catenin) blockade as a resistance-relevant axis even without added chemotherapy [76]. A lipid–copolymer hybrid “BioMIC” platform co-delivered BCL-2 siRNA with the sensitizer quercetin and produced orthotopic tumor regression and fewer lung nodules, though no standard chemotherapeutic was used [23]. Aptamer-functionalized mesoporous silica nanoparticles combining doxorubicin with BCL-2/BCL-xL siRNAs restored sensitivity in resistant 3D cultures, achieving more than 10–40-fold reductions in doxorubicin half-maximal inhibitory concentration (IC_50_) but lacking in vivo confirmation [24].

**Figure 3:**
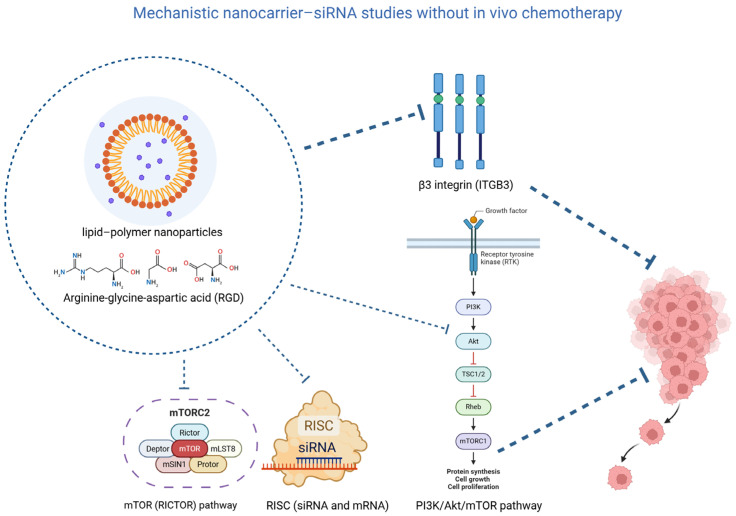
Schematic representation of mechanistic nanocarrier–siRNA systems targeting key molecular pathways involved in tumor growth and metastasis. Lipid–polymer nanoparticles functionalized with RGD peptides deliver siRNA to silence β3 integrin (ITGB3), modulating the PI3K/Akt/mTOR signaling pathway and inhibiting tumor progression. Additionally, siRNA targeting RICTOR downregulates mTORC2 activity, contributing to the suppression of cancer cell proliferation and metastasis.

Additional platforms mapped a broader target space: mesoporous-silica systems delivering polo-like kinase 1 (PLK1) siRNA curtailed metastatic burden with survival benefit in vivo [35]. Arginine-glycine-aspartic acid (RGD)-targeted ECO lipid–polymer nanoparticles silencing β3 integrin (ITGB3) reduced invasion and metastatic dissemination in orthotopic models (Figure 3) [78]. Graphene oxide nanocarriers delivering rapamycin-insensitive companion of mTOR (RICTOR) siRNA inhibited protein kinase B (Akt)/mechanistic target of rapamycin complex 2 (mTORC2) signaling and suppressed xenograft growth [77], while ECO nanoparticles carrying differentiation antagonizing non-protein coding RNA (DANCR) siRNA downregulated EMT/Wnt pathways, achieving significant tumor inhibition in vivo [26].

Several studies remained in vitro but contributed to methodological advances. Forkhead box protein M1 (FOXM1) siRNA nanoparticles synergized with paclitaxel and olaparib in resistant TNBC cells [33], while dual FOXM1/TGF-β1 knockdown in Poly(ethylene glycol)–poly(2-(dimethylamino)ethyl methacrylate (PEG–PDMAEMA) polyplexes reduced EMT markers and migration [39]. EGFR-specific dodecapeptide (GE11)-decorated chitosan nanoparticles loaded with anti-miR-21 impaired colony formation, induced apoptosis, and blocked AKT/extracellular signal-regulated kinase (ERK) signaling [79]. Frizzled class receptor 7 (FZD7)-targeted polymeric nanoparticles delivering β-catenin siRNA suppressed proliferation and stem-like traits in vitro [80].

Other systems combined therapeutic and diagnostic potential. Aptamer–lipid quantum dot carriers silencing BCL-2 and protein kinase C iota (PKC-ι) achieved tumor suppression and enabled imaging in vivo [37]. Folic acid-decorated superparamagnetic iron oxide-trimethyl chitosan (SPION–TMC) nanoparticles co-delivering enhancer of zeste homolog 2 (EZH2) and cluster of differentiation 73 (CD73) siRNAs inhibited tumor growth and partially restored antitumor immunity [81].

Group 2 studies strengthen the biological rationale and demonstrate versatile delivery technologies. However, the absence of in vivo chemotherapy combinations limits direct conclusions about the reversal of chemoresistance.

Across the dataset, lipid-based and lipid-hybrid systems were most common [23,26,33,37], followed by polymeric micelles/polyplexes [33,39] and inorganic or hybrid carriers (mesoporous silica, graphene oxide, nanoshells) [24,35,76,77,78,81].

Frequently targeted pathways aligned with known resistance mechanisms: apoptosis regulation (BCL-2 family) [23,24], Wnt/β-catenin [79], mitotic/kinase signaling (FOXM1, PLK1) [33,35,39], mTORC2 survival signaling (RICTOR) [77], and EMT/invasion programs (DANCR, ITGB3) [26,78]. Emerging platforms also engaged epigenetic and immune axes (EZH2/CD73) [81] and EGFR-related pathways [37,79].

Reporting of tolerability was generally acceptable in animal studies [23,26,35,76,77,78,81], although comprehensive toxicity panels and risk-of-bias elements (randomization, blinding, power calculations) were variably documented. Studies in Group 1, which incorporated standard chemotherapy in resistant TNBC models, demonstrated higher translational relevance, with combined therapy regimens producing more pronounced tumor regression and more robust mechanistic reversal of drug resistance compared to those in Group 2. Group 2 studies, while essential in mapping resistance-associated molecular networks, highlight a persistent gap between preclinical mechanistic feasibility and clinically relevant therapeutic integration.

## 6. Discussion

### 6.1. Beyond Nanocarrier-Delivered siRNA

Preclinical studies suggest that nanocarrier-delivered siRNA can modulate several pathways implicated in TNBC chemoresistance, including efflux transporters [22], DNA repair enzymes [13], anti-apoptotic regulators [23,24], autophagy mediators [74,75], and EMT drivers [26,78], thus directly addressing the polygenic basis of TNBC chemoresistance [18,82].

In our dataset, only eight studies [13,22,34,36,38,73,74,75] tested in vivo co-delivery with standard cytotoxics, yet these were the ones that most consistently produced tumor regression, chemo sensitization, and—importantly in several cases—reduced metastatic burden, underscoring that synergy (siRNA + matched chemotherapy), not substitution, is the translationally relevant principle.

Lipid and lipid-hybrid carriers dominated the translationally relevant space, supported by their consistent in vivo efficacy and established clinical track record [22,36,73]. Polymeric platforms contributed precision through stimuli-responsive release, exemplified by redox-sensitive micelles for AURKA–paclitaxel [38], and dendritic poly-L-lysine–paclitaxel–AXL nanocomplexes [34]. Autophagy inhibition emerged as an additional sensitization mechanism, with exosome- or polymer-based siRNA delivery against DARS-AS1, LC3, and related nodes potentiating anthracycline efficacy [74,75]. Inorganic/hybrid carriers (e.g., porous silicon, graphene oxide, mesoporous silica) offered multifunctionality and drug–gene synchrony [13,76] but will require more comprehensive safety evaluation before clinical advancement.

Despite encouraging preclinical results, each nanocarrier platform faces important translational barriers. Lipid-based systems benefit from prior clinical use and scalable manufacturing but require careful control of batch-to-batch consistency and mitigation of innate immune activation [29,83]. Polymeric carriers offer adaptable architectures and stimuli-responsiveness, yet concerns remain regarding biodegradation kinetics, off-target accumulation, and chronic toxicity [84]. Inorganic and hybrid platforms demonstrate functional versatility, but their long-term biopersistence, clearance mechanisms, and potential organ deposition raise significant safety considerations [85]. Across all systems, regulatory pathways remain complex, as siRNA–nanocarrier combinations are classified as advanced drug–device products requiring detailed characterization of both the nucleic acid payload and the carrier [86]. These challenges highlight that while preclinical data are promising, substantial optimization and rigorous toxicological assessment are required before meaningful clinical translation can occur.

The therapeutic landscape of TNBC has evolved significantly over recent years, but despite breakthroughs, durable control remains elusive—especially in the post-resistance setting [17,87]. In early-stage disease, one clinical trial demonstrated that adding pembrolizumab to neoadjuvant chemotherapy and continuing as adjuvant therapy led to a significant overall-survival benefit: 60-month overall survival (OS) rates were 86.6% with pembrolizumab versus 81.7% with chemotherapy alone (*p* = 0.002; median follow-up 75.1 months) [57].

In metastatic TNBC, other clinical trials established sacituzumab govitecan as a new standard of care, showing superior progression-free survival and OS compared to standard single-agent chemotherapy in heavily pretreated patients [88,89]. Thus, immune checkpoint inhibitors and antibody–drug conjugates (ADCs) now represent therapeutic mainstays across early and metastatic TNBC.

Despite major advances in immunotherapy and ADCs, therapeutic resistance remains a formidable barrier in TNBC [17,87]. One promising approach to address this challenge lies in siRNA nanotherapeutics, which could silence critical mediators of resistance such as MRP1 (drug efflux) [22], RAD51 (DNA repair) [13], AURKA/AXL/IKBKE (mitotic and survival signaling) [34,36,38], Wnt/β-catenin (invasion/EMT) [76], and autophagy [75]. By directly disrupting these pathways, siRNA could resensitize tumors to chemotherapy [13,22,34] and suppresses metastatic dissemination [26,35,78] and potentially prime the TME for improved responses to ADCs or immune checkpoint inhibitors [24].

Evidence from the 21 studies included in this review converges on the principle that co-delivery of siRNA with chemotherapy produces the most robust therapeutic effects. For example, silencing MRP1 in combination with doxorubicin enhanced intratumoral drug retention and significantly suppressed tumor growth in resistant TNBC models [22]. Similarly, a RAD51-targeting siRNA delivered via a porous-silicon/liposome hybrid system restored anthracycline sensitivity, reducing both orthotopic tumor burden and lung metastases [13]. Comparable benefits were observed when siRNAs against AURKA, AXL, or IKBKE were combined with taxane chemotherapy. In one study, AURKA siRNA encapsulated in redox-responsive micelles together with paclitaxel achieved significantly greater tumor suppression than either agent administered alone [38]. Similarly, co-delivery of AXL siRNA with paclitaxel effectively reversed acquired resistance in an orthotopic 4T1/PTX TNBC model, restoring sensitivity to the taxane backbone [34]. In another approach, IKBKE siRNA loaded into folate-targeted liposomes and administered with cabazitaxel resulted in enhanced tumor control and reduced invasiveness, highlighting the therapeutic value of NF-κB pathway inhibition in combination with taxanes [36].

Beyond direct chemo-sensitization, antibody–siRNA complexes targeting the Wnt/β-catenin pathway significantly suppressed tumor regrowth, recurrence, and lung metastasis, even in the absence of concurrent chemotherapy, highlighting their potential as anti-metastatic interventions [76,80].

**Figure 4:**
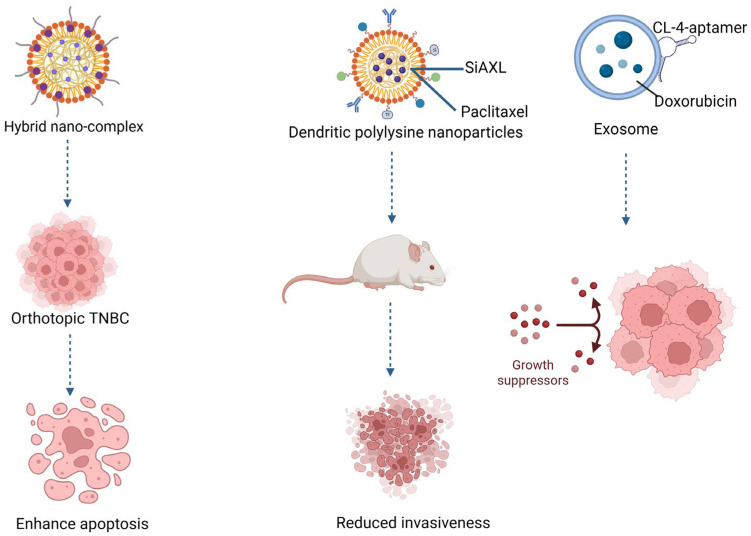
Nanocarrier-delivered siRNA therapeutics. siRNA nanocarriers restore chemotherapy sensitivity in TNBC. Dendritic polylysine nanoparticles carrying siAXL and paclitaxel reversed drug resistance and restored chemosensitivity. CL4-aptamer–engineered exosomes with doxorubicin inhibited autophagy and reduced tumor growth. Together, these siRNA-based nanocarriers overcome resistance via complementary pathways, improving therapeutic response and survival in TNBC models.

Together, these “mechanistic matching” strategies illustrate a clear translational principle: synchronous gene silencing and drug delivery maximizes therapeutic impact, providing durable tumor control and extending therapeutic relevance beyond cytotoxic backbones. Figure 4 summarizes key features of nanocarrier-delivered siRNA therapeutics.

A deeper examination of delivery systems reveals distinct strengths and limitations. Lipid-based nanoparticles and liposomes achieved consistent in vivo efficacy and benefit from precedents in clinical translation (e.g., LNPs for mRNA vaccines), though their biodistribution remains liver-biased and safety reporting heterogeneous [13,22,36,73]. Polymeric carriers provided tunable, stimuli-responsive designs, such as GSH-triggered release with redox micelles [74], or dendritic poly-L-lysine for AXL–paclitaxel co-delivery [39]. Inorganic and hybrid platforms offered multifunctionality: mesoporous silica nanoparticles carrying PLK1 siRNA improved survival [35]; graphene oxide delivering RICTOR siRNA curtailed AKT signaling [77]; and porous-silicon/liposome hybrids improved drug–gene synchrony [13]. Nevertheless, their long-term safety and biodegradability remain incompletely defined.

Mechanistic attribution requires caution because improved outcomes can arise from (i) enhanced drug delivery/retention driven by the carrier rather than target biology, (ii) off-target gene silencing or innate immune activation, and (iii) toxicity-induced reductions in tumor volume that mimic efficacy. Accordingly, we interpret pathway claims as strongest when studies jointly demonstrate target knockdown at mRNA/protein levels, a functional downstream readout consistent with the pathway (e.g., increased intracellular drug accumulation for efflux targets; increased γH2AX/apoptosis for DNA repair targets), and appropriate controls (scrambled siRNA, empty carrier, and chemotherapy-alone arms). Where these elements were absent or incompletely reported, conclusions are framed as supportive but not definitive.

### 6.2. Challenges and Optimization Strategies

The dense extracellular matrix and aberrant vasculature of TNBC present significant barriers to nanocarrier penetration. Strategies exploiting RGD-mediated integrin targeting have demonstrated improved delivery efficiency and reduced invasion and metastatic dissemination [26,78]. Likewise, multistage porous silicon–liposome hybrids coordinated sequential drug and siRNA release, achieving deeper tissue penetration and synergistic antitumor effects [13].

Nevertheless, rapid clearance by the reticuloendothelial system and innate immune activation remain critical challenges. Although most studies reported acceptable tolerability, comprehensive toxicology—including hematological parameters, complement activation, long-term organ deposition, and immunogenicity—was inconsistently assessed, particularly in the case of inorganic platforms [35,77,81]. To mitigate these risks, design improvements such as PEGylation, optimization of particle size and shape, and incorporation of tumor-specific ligands have been explored. Examples include IKBKE–cabazitaxel hybrid nanocomplexes with enhanced therapeutic activity [36], functionalized siRNA–chitosan nanoparticles that disrupted the miR-21/AKT/ERK axis with tumor selectivity [79], and aptamer-directed nanocarriers enabling receptor-specific delivery [37].

Data across studies converge on three design principles: (a) Co-delivery of cytotoxic agents with siRNA against a mechanistic resistance driver (e.g., MRP1/DOX [22]; RAD51/DOX [13]; IKBKE/cabazitaxel [36]; AXL/PTX [34] to produce synergistic resensitization; (b) Stimulus-responsive release (redox, ultrasound) to enhance intracellular bioavailability at the disease site [38,90]; and (c) Ligand-guided targeting (folate, RGD, EGFR aptamer/GE11) to increase tumor-cell specificity and reduce normal-tissue exposure [36,37,78,79].

Overall, the included studies provide encouraging but preliminary support for siRNA-based approaches to overcoming TNBC chemoresistance. However, variability in experimental designs, resistance models, and delivery platforms limits the generalizability of these findings. Thus, any extrapolation to clinical practice should remain cautious until more rigorous and standardized in vivo evidence becomes available.

Methodological reporting was variable across studies, particularly regarding randomization, blinding, and power calculation. Importantly, only a subset of studies rigorously validated chemotherapy-resistant phenotypes before claiming re-sensitization. Accordingly, findings derived from validated resistant orthotopic models with appropriate comparators are interpreted as stronger evidence of chemo-re-sensitization, whereas studies lacking resistance validation or detailed bias-mitigating measures are interpreted primarily as proof-of-mechanism. These considerations support a cautious framing of translational readiness.

### 6.3. Future Perspectives

Only a subset of studies used orthotopic or resistant models (e.g., 4T1/PTX for AXL siRNA) or 3D spheroids (aptamer-MSNP BCL-2/BCL-xL), yet these produced the most translationally instructive results [24,34]. Future work should emphasize patient-derived xenograft (PDX) and immunocompetent models and report standardized pharmacokinetics/toxicity.

Rational pairing of targets with drugs, MRP1 anthracyclines; RAD51 anthracyclines/platinum; AURKA/AXL taxanes; IKBKE taxanes; Wnt/β-catenin or DANCR metastatic control, is already supported by the included datasets and should be advanced into dose-finding and schedule-optimization studies [13,22,34,36,38,76].

Artificial intelligence (AI)-guided materials screening and multi-objective optimization (encapsulation, endosomal escape, TME penetration, toxicity) could shorten iteration cycles. Although these tools were not directly tested in the included studies, they are well-suited to integrate physicochemical design spaces with in vivo readouts, the field now reports. The preclinical literature supports the biological plausibility that siRNA can modulate resistance pathways in TNBC and, in a subset of studies, improve chemotherapy response. However, the evidence base remains heterogeneous with respect to model selection, resistance validation, dosing comparability, and safety reporting. We therefore distinguish proof-of-mechanism (target knockdown and pathway modulation with tumor control) from translational readiness, which additionally requires reproducible manufacturing, defined product specifications (size distribution, encapsulation, stability), clinically relevant biodistribution and tumor exposure, immunogenicity/complement assessment, clearance pathways, and repeat-dose toxicology. Most included studies provide early efficacy signals but do not yet meet the breadth of data typically required to support clinical development decisions.

### 6.4. Limitations

This narrative review relied primarily on the PubMed database and did not follow a preregistered systematic-review protocol. Therefore, it may have missed studies indexed in other databases. Although we did not restrict language during database searching, inclusion required an accessible English full text to enable standardized extraction and appraisal of experimental methods and outcomes; therefore, potentially relevant non-English full texts may have been missed. We performed a structured appraisal of key internal validity and reporting domains (randomization, blinding, resistance validation, endpoint reporting), but this was not a formal tool-based risk-of-bias assessment (e.g., SYRCLE) with study-level scoring; therefore, residual bias cannot be excluded. As a result, the robustness of individual experimental designs and potential sources of bias were not systematically evaluated. Therefore, comparative or translational interpretations should be viewed cautiously, and future systematic reviews applying validated risk-assessment tools are warranted. The studies included in Group 2 varied in their use of resistance models, and several did not confirm a resistant phenotype or incorporate chemotherapy co-administration, which may limit the direct translational interpretation of their findings.

## 7. Conclusions

The siRNA nanocarriers evaluated in the included studies demonstrated the ability to modulate resistance-related pathways and, in several cases, enhance chemotherapy efficacy in TNBC models. Some platforms also reported effects on metastatic endpoints. Among the delivery systems studied, lipid and lipid-hybrid carriers showed the most consistent combination of delivery performance and translational plausibility, whereas polymeric constructs provided stimuli-responsive precision and inorganic or hybrid systems offered functional versatility, though these latter categories will require more extensive safety characterization.

Although these findings are encouraging, they remain preliminary and heterogeneous. A potential direction for future work is the further exploration of co-delivery strategies that pair standard chemotherapeutic agents with mechanistically relevant siRNAs. Such approaches would benefit from evaluation in well-validated resistant orthotopic or patient-derived xenograft models, using standardized pharmacological and toxicity assessments. Additionally, stimuli-responsive and ligand-targeted designs may help address current limitations related to tumor penetration and cellular uptake. Approaches such as computational or AI-assisted carrier optimization could also support the rational refinement of delivery architectures. Collectively, the targets most frequently investigated across the reviewed studies—MRP1, RAD51, AURKA, AXL, IKBKE, β-catenin/Wnt, DANCR, PLK1, and ITGB3—highlight mechanistic nodes that warrant further preclinical exploration, while underscoring the need for more rigorous evaluation before any movement toward early-phase clinical investigation in chemoresistant TNBC can be considered. Overall, siRNA nanocarriers show encouraging preclinical proof-of-mechanism for modulating chemoresistance pathways in TNBC, and a smaller subset demonstrates in vivo chemo-combination benefit in models that variably validate resistance. These findings justify further optimization and standardized testing rather than direct inference of near-term clinical applicability.

## Figures and Tables

**Table 1:** siRNA-loaded nanocarriers overcoming chemotherapy resistance in TNBC (Group 1 studies).

Reference	Nanocarrier Type	siRNA Target	Chemotherapy	Model	Resistant Phenotype Explicitly Validated	Key Outcome
Deng (2013) [22]	Layer-by-layer lipid–polymer hybrid nanoparticles	MRP1	Doxorubicin (DOX)	MDA-MB-231 xenografts	Partial (efflux mechanism targeted; no stable resistant subline described)	Restored DOX sensitivity; ~8-fold tumor volume reduction vs. control.
Yin (2015) [38]	Redox-sensitive HA-based micelles (HSOP)	AURKA	Paclitaxel (PTX)	MDA-MB-231 xenografts	No (no formal resistant phenotype validation)	Synergistic in vivo tumor inhibition; improved delivery and accumulation.
Zhao (2020) [36]	HA-modified hybrid nanocomplex	IKBKE	Cabazitaxel	Orthotopic TNBC	No (resistance inferred; not formally validated)	Synergistic tumor inhibition and survival benefit vs. single agents.
Wan (2024) [34]	HA-modified dendritic poly-lysine nanoparticles	AXL	Paclitaxel (PTX)	PTX-resistant 4T1 orthotopic TNBC	Yes (validated PTX-resistant 4T1/PTX model	Restored paclitaxel sensitivity in resistant tumors; significant regression.
Bhullar (2024) [73]	CD44-aptamer engineered extracellular vesicles	Survivin	Gemcitabine + Paclitaxel (PTX)	Orthotopic TNBC	No (dose-reduction strategy; no resistant model validated)	Achieved major chemo dose-reduction with preserved efficacy; lower toxicity.
Liu (2023) [74]	CL4-aptamer exosomes	lncRNA DARS-AS1	Doxorubicin (DOX)	TNBC xenografts	Partial (anthracycline resistance context; limited validation detail)	Inhibited autophagy; reversed DOX resistance; stronger tumor suppression.
Walweel (2025) [75]	Polymeric smart nanoparticles	LC3	Doxorubicin (DOX)	TNBC xenografts	Partial (autophagy-linked resistance)	Autophagy suppression restored DOX efficacy; significant tumor inhibition.
Wu (2021) [13]	Porous silicon microparticles encapsulating DOPC liposomes with siRad51	Rad51	Doxorubicin (DOX)	Orthotopic TNBC and lung metastasis mouse models	Yes (DNA repaird-mediated resistance mechanism validated)	Combined therapy significantly reduced primary tumor burden and lung metastasis, overcoming DOX resistance.

Although all studies in Group 1 evaluated in vivo chemo-combination strategies, only a subset explicitly validated stable chemotherapy-resistant phenotypes prior to combination testing. In several cases, re-sensitization was inferred from improved combination efficacy relative to chemotherapy alone without formal demonstration of a resistant subline or sustained IC_50_ shift. These distinctions are indicated in Table 1 and were considered when interpreting translational strength.

**Table 2:** Group 2—siRNA nanocarriers in TNBC with mechanistic or synergistic effects (without explicit in vivo chemotherapy re-sensitization).

Reference	Nanocarrier Type	siRNA Target/Payload	Chemotherapy	Model	Key Outcome
Vaidya (2019) [26]	ECO lipid-polymer nanoparticles	DANCR (lncRNA)	—	Orthotopic TNBC xenografts	Significant in vivo tumor growth suppression by systemic siRNA delivery.
Yang (2021) [77]	Graphene oxide nanoparticles	Rictor (mTORC2)	—	MDA-MB-231 xenografts	Effective siRNA delivery and tumor suppression in vivo.
Misra (2021) † [33]	Polymeric nanoparticles	FOXM1 + Paclitaxel + Olaparib	Paclitaxel (PTX) + Olaparib	In vitro (MDA-MB-231)	Synergistic effects demonstrated in vitro; no in vivo validation.
Dang (2024) [76]	Silica–gold nanoshells (FZD7 antibody + siRNA)	β-catenin (Wnt)	—	Orthotopic + metastasis models	Suppressed tumor growth and lung metastasis in vivo.
Li (2023) [23]	Lipid–copolymer hybrid nanocomplex (BioMICs)	BCL-2 siRNA + Quercetin	—	Orthotopic 4T1 TNBC + metastasis	Regression of tumors and lung metastases in vivo.
Kumar (2023) † [24]	Aptamer-functionalized mesoporous silica nanoparticles (MSNPs)	BCL-2, BCL-xL + DOX	Doxorubicin (DOX)	Resistant MDA-MB-231 (3D in vitro)	>10–40× reduction in DOX IC_50_; strong in vitro chemo re-sensitization, no in vivo.
Morry (2017) [35]	Antibody-targeted MSNP-PEI-PEG	PLK1	—	TNBC lung metastasis model	~80% PLK1 knockdown, reduced metastatic burden, improved survival.
Parvani (2015) [78]	RGD-targeted ECO lipid–polymer nanoparticles	ITGB3 (β3 integrin)	—	TNBC xenografts, metastasis models	Inhibited EMT and metastasis in vivo.
Wang (2024) [39]	Disulfide crosslinked PEG-PDMAEMA nanoparticles	TGF-β1, FOXM1	—	In vitro (MDA-MB-231, MG-63)	Suppressed EMT, migration, invasion; no in vivo validation.
Abdulmalek (2024) [79]	GE11-peptide–chitosan nanoparticles	miRNA-21	—	In vitro (MDA-MB-231)	Reduced migration/colony formation, induced apoptosis, blocked AKT/ERK signaling.
Hoover (2025) [80]	FZD7-targeted polymer nanoparticles	β-catenin (Wnt)	—	In vitro	Suppressed stem-like phenotypes, proliferation, and drug resistance traits.
Kim (2019) [37]	Aptamer-conjugated lipid nanoparticles + quantum dots	Bcl-2, PKC-ι siRNAs	—	MDA-MB-231 xenografts	In vivo tumor growth/metastasis inhibition and imaging capability.
Adibfar (2022) [81]	Folic acid-functionalized SPION-TMC nanoparticles	EZH2 + CD73	—	In vitro and in vivo TNBC	Tumor regression and restored antitumor immune responses.

† These studies combined siRNA nanocarriers with chemotherapeutics and demonstrated strong chemo re-sensitization in vitro but lacked in vivo validation. They were included to illustrate innovative strategies, although their translational relevance is limited compared to in vivo studies.

## Data Availability

No new data were created or analyzed in this study.

## Abbreviations

4T1/PTX	4T1 mouse mammary carcinoma cell line treated with paclitaxel
ABC	ATP-binding cassette
ABCB1	ATP-binding cassette sub-family B member 1 (or MDR1)
ABCG2	ATP-binding cassette subfamily G member 2
ADCs	antibody–drug conjugates
Ago2	argonaute-2
AI	artificial intelligence
AURKA	aurora kinase A
AKT	protein kinase B
AXL	tyrosine kinase receptor
BCL-2	B-cell lymphoma-2
BRCA1	breast cancer gene 1
BRCA2	breast cancer gene 2
CD44	cluster of differentiation 44
CD73	cluster of differentiation 73
DANCR	differentiation antagonizing non-protein coding RNA
DARS-AS1	aspartyl-tRNA synthetase antisense RNA 1
DOX	doxorubicin
EGFR	epidermal growth factor receptor
EMT	epithelial-to-mesenchymal transition
ER	estrogen receptor
*ERCC1*	excision repair cross-complementation group 1
EZH2	enhancer of zeste homolog 2
ERK	extracellular signal-regulated kinase
FOXM1	forkhead box protein M1
FZD7	frizzled class receptor 7
GE11	EGFR-specific dodecapeptide
HER2	human epidermal growth factor receptor 2
IC_50_	half-maximal inhibitory concentration
IKBKE	inhibitor of nuclear factor kappa-B kinase epsilon
ITGB3	β3 integrin
KRAS	Kirsten rat sarcoma viral oncogene homolog
LC3	microtubule-associated protein 1 light chain 3
LNPs	lipid nanoparticles
MAPK	mitogen-activated protein kinase
MCL1	myeloid cell leukemia-1
MDR1	multidrug resistance protein 1
mRNA	messenger RNA
MRP1	multidrug resistance-associated protein 1
MSNP	mesoporous silica nanoparticle
mTOR	mechanistic target of rapamycin
mTORC2	mechanistic target of rapamycin complex 2
MYC	myelocytomatosis
NF-κB	factor nuclear kappa B
OS	overall survival
PDX	patient-derived xenograft
PTX	paclitaxel
*PARP1*	poly(ADP-ribose) polymerase 1
PDMAEMA	poly(2-(dimethylamino)ethyl methacrylate
PEG	poly(ethylene glycol)
PI3K	phosphatidylinositol 3-kinase
PKC-ι	protein kinase C iota
PLK1	polo-like kinase 1
PR	progesterone receptor
RAD51	radiation-sensitive protein 51
*RAS*	rat sarcoma
*RB1*	retinoblastoma 1
RGD	arginine-glycine-aspartic acid
RICTOR	rapamycin-insensitive companion of mTOR
RISC	RNA-induced silencing complex
RNAi	RNA interference
siRNA	small interfering RNA
Smad3	Sma and Mad 3
SPION-TMC	superparamagnetic iron oxide-trimethyl chitosan
TME	tumor microenvironment
TNBC	triple-negative breast cancer
*TP53*	tumor protein p53
TGF-β	transforming growth factor-β
TSGs	tumor suppressor genes
Wnt/β-catenin	Wingless and int-1/β-catenin
